# A qualitative study using hybrid simulation to explore the impacts of human factors e-learning on behaviour change

**DOI:** 10.1186/s41077-020-00136-y

**Published:** 2020-08-12

**Authors:** Harry Carter, Sally Hanks, Thomas Gale

**Affiliations:** 1 University College London Hospital, London, UK; 2 University of Plymouth, Plymouth, UK; 3 Peninsula Medical School, Portland Square, University of Plymouth, Drake circus, Plymouth, PL4 8AA UK

**Keywords:** E-learning, Human factors, Hybrid simulation, Undergraduate, Clinical skills, Communication skills

## Abstract

**Background:**

There is an international drive to increase human factors training in undergraduate medical curricula through various educational platforms. E-learning can be effective at teaching technical skills but there is limited research exploring the benefits of e-learning in human factors training. This study aimed to utilise hybrid simulation to investigate the impact of a human factors focused e-learning package for intravenous cannulation on safety behaviours.

**Methods:**

Video-reflexive ethnography (VRE) techniques and interviews were used to explore human factor-related behaviour change in hybrid simulation scenarios, before and after e-learning modular training. Ten final-year medical students were recruited for the study. Content analysis of VRE data from hybrid simulation scenarios identified which behaviours had changed; thematic analysis of semi-structured interviews uncovered why.

**Results:**

Results demonstrate improvement in safety behaviours in the domains of physical-, cognitive- and macro-ergonomics, suggesting safer cannulation practice following training. Online videos with interactive activities were reported as the major pedagogical driver for change. The impact of the e-learning was identified across four themes: environment, person, policy-related tasks, and preparedness for practise. Reported intention to change practise and altered behaviour in the workplace supports the conclusion that this training prepares students for practise by facilitating them to incorporate human factors principles in their clinical work.

**Conclusion:**

E-learning is a valuable and effective method for supporting medical student learning about human factors. Hybrid simulation and VRE combine well together to evaluate behaviour change following educational interventions.

## Background

The application of human factors in the workplace is recognised as a key component of safe practise [1] and is a recommended outcome for medical graduates and doctors [2, 3]. Doctors are expected to display a wide range of non-technical skills and safety behaviours in order to work effectively in teams, recognise their own limitations and reduce risks to patients. Training in human factors has been identified as a valuable topic to deliver in undergraduate medical curricula by the World Health Organisation in their Patient Safety Curriculum for medical students [4], enabling the reliable acquisition of Crisis Resource Management (CRM) skills and behaviours which lead to safe practise [5]. Simulation has been shown to be particularly effective for training in CRM principles and non-technical skills but there are significant resource implications of embedding simulation into an undergraduate medical curriculum [6, 7].

Within these constraints, Norman suggested use of the term *Simulation-Augmented Medical Education* rather than *Simulation-Based Medical Education* in order to fully embed simulation and human factors training into curricula alongside other educational methods [7]. E-learning has been combined successfully with simulation to reduce the overall length of traditional courses such as advanced cardiac life support run by the American Heart Association [8], and has been utilised for specific training in non-technical skills [9, 10], but research evaluating its use for human factors and ergonomics training is still lacking. E-learning holds some advantages over other educational methods since e-learning can engage numerous learners with standardised content, increase accessibility of training and lower long-term costs [11].

Hybrid simulation utilises multiple simulation modalities to increase fidelity when procedural skills are being performed [12]. This is commonly created with a standardised patient (SP) wearing a Part-Task Trainer (PTT) so that the learner can be assessed interacting with challenges related to the physical task as well as the interpersonal or human factors involved [13, 14]. Hybrid simulation is increasingly used for assessment of a combination of procedural and non-technical skills but could also be utilised as a method for assessing learning outcomes from other educational modalities providing training in these skills. To assess human factors, simulation needs to provide a realistic environment, person-person interaction, relevant tools, appropriate challenge and a measurable outcome.

Video-reflexive ethnography (VRE) is a research method that facilitates analysis and reflection of individuals involved in an activity. Clinicians watch videos of themselves undertaking routine tasks followed by opportunities to discuss and reflect on the situation. The theoretical premise of VRE is that clinicians inhabit and work within a ‘zone of maximum complexity’ [15] day in, day out and this is not accessible to an observer or through analysis of reported or recalled data alone. Use of VRE can lead to transformative learning via insight provided by participants through reflexivity and interpretation of their activities while undertaking clinical work in this zone [16–18]. The VRE technique embeds the participant’s meaning, i.e. the interpretative naturalistic enquiry element, into research findings. This study aimed to explore the potential impacts of human factors e-learning on medical students’ safety behaviours in hybrid simulation scenarios using VRE techniques.

## Methods

### Procedures

**Fig. 1:**
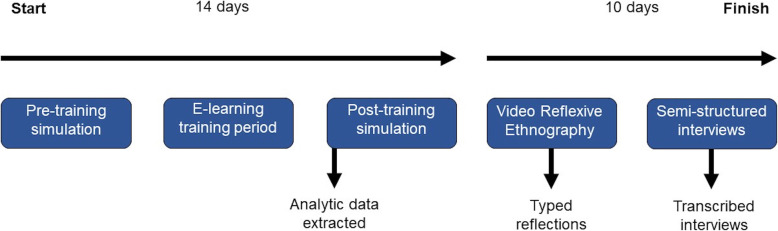
Schematic of the study design and timeline

Participants were recruited and consented to take part in the study and then allocated two simulation time slots, 2 weeks apart, where performances would be recorded by video. For the first simulation scenario, each student would be familiarised with the simulation set-up and allowed 5 min to read a vignette prior to taking part. Following the first simulation, students were given access to the e-learning platform for 2 weeks and asked to complete the human factors module designed for intravenous cannulation. Students then completed a second scenario which was set up in a similar way as the first scenario. Thereafter, participants were provided with personal simulation recordings from both scenarios to review. A VRE template for both scenarios, with written instructions on how to complete it, was provided to the students to compare their performance between the two scenarios. Semi-structured interviews were conducted with all students by author HC after the VRE templates had been completed (see Fig. 1).

### Design

Qualitative methodologies were employed to explore behaviour change following engagement with the e-learning intervention. Pre- and post-training simulation exercises were undertaken to support investigation of a range of human factor-related issues when performing intravenous cannulation. This method was chosen because relevant educational studies have used pre- and post-testing with good results [19, 20], and there is no standardised way to quantify human factors behaviours.

### Hybrid simulations

The simulation design consisted of a SP wearing a specialised cannulation sleeve (PTT) to mimic cannulation on a real patient. This facilitated realistic communication [13], integration of skills and adaptation of scenarios [12]. Utilising the university clinical skills area, we recreated a ward environment with a bed, nurses’ station, patient notes and equipment trolley. Two white-British, middle-aged actors (one for each afternoon) and a student nurse were recruited. Briefing details were sent to all actors in advance, and they were verbally briefed on the day, before changing into patient gowns and nursing uniform. There was no script, but clear instructions were given for how to react at specific times during the scenario. The actor then applied the cannulation sleeve. Participants arrived for their time slot and read their brief, before entering the simulation, which lasted between 10 and 22 min. The scenarios and SPs used in the pre- and post-training simulations differed, to reduce anticipation of challenges by participants, reduce standardisation and enhance complexity, so that the second scenario also provided an unfamiliar environment for the students. In each scenario, students had to manage a range of human factor-related issues, including poor lighting and equipment layout, unfamiliar tools, challenging patient positioning and patient distress.

The cannulation sleeve was made from a leather and Kevlar wrist plate for protection from needlestick injuries, plastic tubing attached to a 500-ml bag of simulation blood and a silicone sleeve fashioned from the ‘skin’ of a venipuncture model, held together with Velcro strips. Issues that we encountered with the simulation included dilution of fake blood with flushes, consistency of the plastic tubing (initially too firm) and leakage if the tube was overused. These issues were corrected between scenarios. Other limitations to the sleeve included a single ‘vein’ to cannulate on the forearm, leaving no room for choice of cannulation site.

### E-learning intervention

**Fig. 2:**
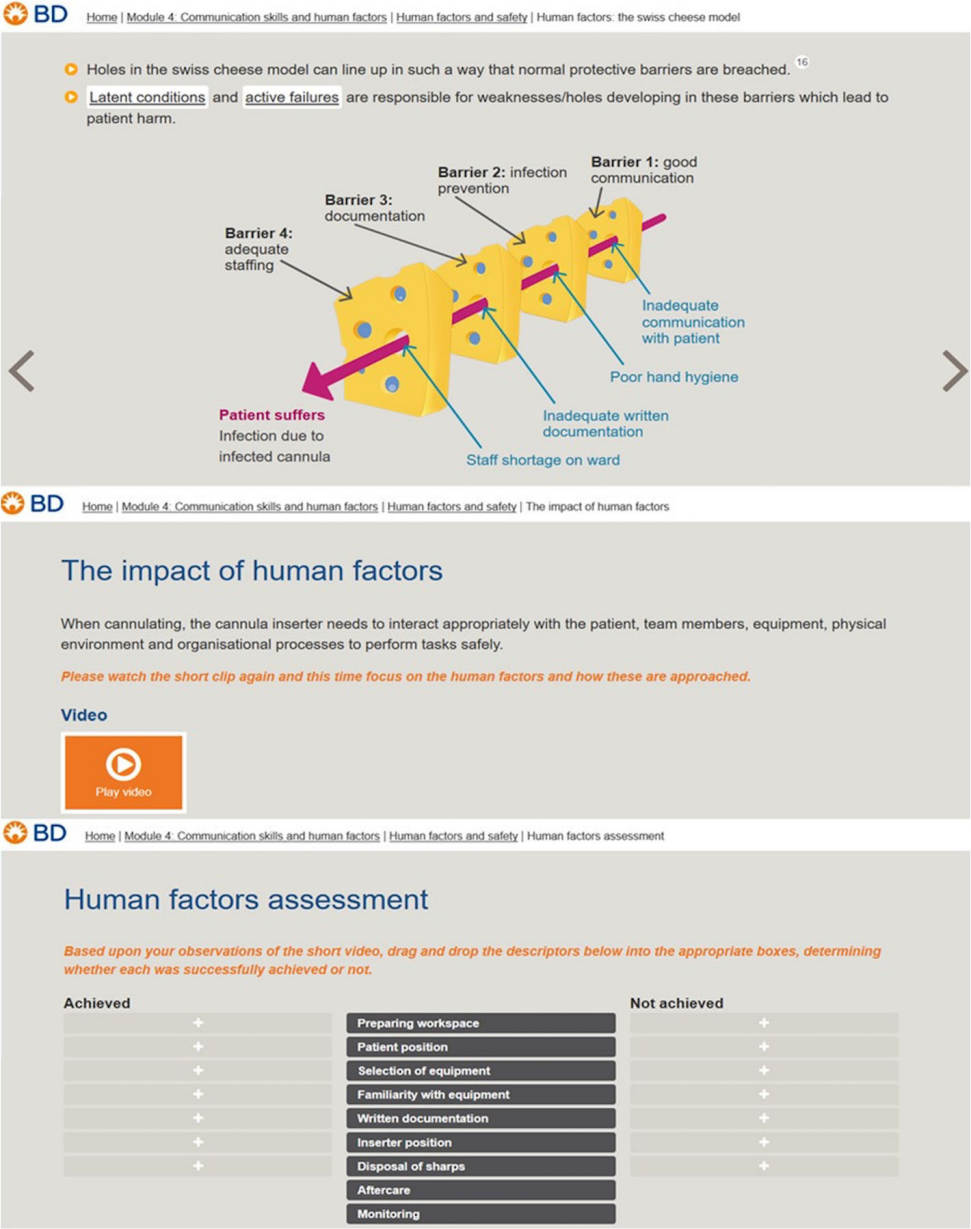
Screenshots from the ‘Communication Skills and Human Factors’ module, showing relevant theory [18], demonstrative video and retrospective ‘drag and drop’ activity

An e-learning resource was created in a collaboration with clinical academics at the University of Plymouth and vascular access product specialists Becton Dickinson (BD). The resource relates to advanced peripheral intravenous cannulation and is split into four modules (see Table 1). The fourth module utilised human factors theory (including the Swiss Cheese Model) [21], demonstrative videos (showing positive and negative safety behaviours) and retrospective analysis activities in the form of ‘drag and drop’ quizzes dispersed throughout the module. The Systems Engineering Initiative for Patient Safety (SEIPS) model [11] is a widely accepted and applied model of healthcare sociotechnical systems, used to classify ‘safety behaviours’ relevant to human factors and promote understanding of how practitioners interact with different parts of the work system, the SEIPS model was embedded into this e-learning tool, within the context of performing peripheral cannulation. This allowed learners to observe human factors in action and contextualise the principles within a cannulation procedure (see Fig. 2).

**Table 1:** E-learning resource modular structure and learning outcomes. The study focused on evaluating Module 4, ‘Communication Skills and Human Factors’

Prepare	Procedure	Preserve	Communication Skills and Human Factors
• Professional and legal considerations	• Preparing for insertion	• Monitoring the cannula site	• Why communicate?
• Anatomy and physiology	• Steps for peripheral line insertion	• Care and maintenance	• Communication skills
• Assessing your patient	• Unsuccessful cannulation	•Patency	• Human factors and safety
• Cannula selection	• Safety and risk mitigation	•Flushing	• Best practises in communication
		• Removal	
		• Post-procedural complications

### Sampling

Ten final-year medical students from University of Plymouth were recruited as case studies through advertisement on social media pages, forming a self-selected convenience sample. There was no financial incentive, but participants benefited from cannulation training (preparing for junior doctor role), challenging simulation exposure and a certificate for portfolios. The students had already been trained to cannulate in their second year on PTTs and may have had limited opportunities to practise during clinical placements. This sample size was chosen to optimise theoretical data saturation for thematic analysis of the qualitative data [22]. Final-year students were selected because they were preparing for foundation year one (F1) posts, which require the ability to perform cannulation independently as a newly qualified doctor [23].

### Data collection

Data was collected from three sources: (i) analytic data from the e-learning platform, (ii) written VRE reflections and (iii) audio recordings of semi-structured interviews. Analytic data from the e-learning platform was extracted shortly before the post-test simulations. This included the average duration of time online, the frequency with which modules were accessed and participant gender. This data was collected to triangulate self-assessed behavioural change with resource engagement and participant demographics. Using the premise of VRE methodology, students were asked to review their video performances and complete a reflective template based on the systems of the SEIPS model [24] (see Appendix 1). This helped participants to scaffold reflection towards human factors domains and identify ‘what’ had changed from pre- to post-test simulations, giving them insight into their own practice and opportunity to note changes [14, 15]. Following VRE and within 10 days of the second simulation, each participant attended a 15 to 25-min semi-structured interview. These included some a priori questions, which aimed to investigate the educational value of the e-learning resource through the exploration of ‘why’ the identified behaviours changed.

### Data analysis

VRE reflections were analysed using the SEIPS model themes to summarise and code highlighted behaviours and areas of behaviour change [24]. This enabled an overview of ‘what’ areas of human factors had changed. Interviews were typed as verbatim transcriptions, with pauses and fillers for accuracy [25–27], by the lead researcher (HC) to optimise data immersion. Transcripts underwent a thematic analysis to understand ‘why’ behaviours had changed. Data was managed using the NVivo© 11 software. All researchers (HC, TG, SH) reviewed and discussed emerging findings, reducing single-researcher bias and increasing trustworthiness of findings [28].

## Results

### E-learning platform analytic data

Five female and five male students took part in the study, and each participant completed the process. All students finished the mandatory human factors module but engagement with the other three modules varied. The average (mean) time spent on the e-learning resource by participants was 148.1 min, and the average number of times that students accessed any resource module was 10.9. This suggested high engagement with the e-learning resource, with revisiting of modules. The demonstrative videos and associated interactive activities were frequently referred to as a major driver for change in behaviour.

*‘I like seeing videos about the good way to do things as you can copy that and amend it as you see fit.’*

*‘You learn about it (cannulation) and then have to do the quiz, which really made you concentrate and take it in. So I think the setup of that and the little activities as you go along really made you put it into practise.’*

### Content analysis: behaviours and behaviour change

A broad range of behaviours changed from the pre- to the post-training simulation. Content analysis of these as identified from analysis of the reflective templates using the SEIPS model for themes is presented in Table 2.

**Table 2:** Content analysis of behaviours identified through VRE of simulated performances, mapped to the SEIPS model systems

System	Altered behaviour
Environment	Lighting utilised Organise workspace Patient/inserter positioning Gather equipment before disturbing the patient
Tools	Equipment familiarity Kit checks before procedure Tourniquet use Where to look for ‘flashback’ (a sign showing venous access)
Tasks	Clean site Documentation Dressing application Patient identification check Successful cannulation
Person	Clearer instructions Concerns explored Empathy Giving monitoring information Nurse interaction Patient interaction
Organisation	Check saline expiry date Followed cannulation guidelines Informed consent Apologised on behalf of the team High number of cannulation attempts

Overall, there was an improvement in human factor-related behaviours, relating to environmental considerations, equipment familiarity, ability to complete the task, communication and following policy. For example:

*‘In simulation 1 although I gathered all the necessary equipment there was a delay in getting the trolley to use as a base for my equipment. In the second simulation, I retrieved the trolley from the start and this made organising my workspace much better.’*

*‘I only documented on the dressing in the first sim; the second time, I documented both on the dressing and in the notes.’*

*‘Compared to the first simulation I feel that I had improved in my communication skills. I feel that I gained consent and highlighted the importance of monitoring the cannula site to the patient – something that I didn’t do in simulation 1.’*

Some participants noted a consistency in behaviours across both simulations while a small number noted a decline or omission of certain behaviours in the second case. Where a negative change in behaviour was reported, this was often accompanied by increased awareness of such suboptimal activity or attributed to a conscious decision rather than a mistake. For example:

*‘In both scenarios, I feel I could have made more use of lighting available. I was concerned about waking the patient more as he was tired.’*

*‘In the first scenario I turned the lights on as the room was very dark, I did not do this in the second scenario as the room was much lighter.’*

When triangulating behaviour change with resource engagement data, the overall improvement in safety behaviours correlates with high engagement with the resource. This suggests that the e-learning package was a driver for positive behavioural change.

### Thematic analysis: drivers for activity and change

Four themes were identified through analysis of the transcribed interviews relating to how and why human factors thinking and behaviours occurred. Table 3 summarises these themes with their associated behaviour changes.

**Table 3:** Themes relating to reasons for behaviour and changes following training

Environmental	Person	Policy-Related Tasks	Preparedness for Practise
• Prepare equipment	• Patient rapport	• Detailed documentation	• Reinforce basic safety steps
• Patient and inserter positioning	• Patient empowerment	• Expiry date checks	• Hazard and harm awareness
• Optimise lighting	• Giving information	• Altered practise on the wards	• Procedural confidence
	• Team involvement		

#### Theme one: environmental

A major impact of the resource related to participants taking control of environmental human factors more consciously. Organisation of workspace, appropriate patient and healthcare worker positioning and adequate lighting were all factors of safety behaviour that were more explicitly considered following training.

*‘(I learnt about) being aware of the environmental factors, so turn the light on, prepare the equipment, be ready to cannulate.’*

*‘(In the post-training simulation) I moved the trolley next to me to dispose of the sharps safely and have my gauze and equipment ready.’*

Some participants demonstrated enhanced awareness and intention to change areas of their practise.

*‘Positioning-wise, I think that’s something I can change to my practise.’*

Where participants were not familiar with treating patients at night, they reported feeling conflicted about using lighting for fear of disturbing the patient. After completing the simulations and e-learning, they demonstrated more consideration of this aspect and awareness of the need to recognise and manage this conflict and uncertainty. Hence, there was variation in the approaches taken to enable adequate vision.

*‘I had never really considered lighting before… As a student, you are not really there during the night… Turning a light on might agitate a patient but it is important for you to see what you are doing.’*

#### Theme two: person

The interviews demonstrated the impact of the e-learning on interactions with other people within the system, including the patient and the nurse. The resource highlighted communication techniques; some students made this the focus of their post-training simulation.

*‘One of the things highlighted in the resource was how having a cooperative patient who is ready to relax and is comfortable can reduce the amount of errors.*’

*‘In the second (simulation)… in the forefront of my mind was more about communicating to the patient.’*

The e-learning emphasised empowerment of patients to take control of their care. In context, this means giving patients’ information about their cannula, how to care for it and monitor for signs of infection. Furnished with this knowledge, students demonstrated improved patient empowerment in the second simulation with intentions to take this forward into practise.

*‘I remember it (e-learning) mentioning... that patient cooperation, empowering the patient and educating them can help to catch those errors which healthcare team members might miss.’*

*‘I am going to be more conscious of trying to empower the patient.’*

The human factors module made the benefits of effective teamwork clear, leading to greater engagement with the nurse in some instances.

*‘Involving extra team members… you’re going to take a little bit extra pressure off yourself… It’s about utilising team member’s skills and their experience where yours might be lacking.’*

#### Theme three: policy-related tasks

The process had clear impacts on policy-related tasks, including documentation and safety checks. Participants reflected that documentation is an easy step to forget because they seldom complete it as students. Few participants in our sample were aware of the importance of documenting the serial number of the cannula, which was highlighted by the resource.

*‘I’m not used to doing it on the wards because I feel like the doctors take care of it.”*

*“I wrote down the serial number of the cannula for the first time.’*

Change of practise was described as continuing into in the workplace which was positively reinforced by nurse feedback.

*‘I think the resource really highlighted getting the colour and size and number of the cannula (recorded) because often we see in practise when that isn’t documented on the drug charts. The nurses have been giving us positive feedback because we have tried to document it a bit more… so I’m making a conscious effort to make sure it’s documented on the drug chart.’*

Some students improved their safety checks from pre- to post-training simulations, including checking expiry dates and patient identification. This was one of the areas in which demonstrative videos were reported to be useful.

*‘The video went through double checking the flush and expiry date.’*

#### Theme four: preparedness for practise

Students became more aware of the hazards of a ‘simple’ procedure such as cannulation, leading them to think more about patient safety and the impacts of invasive procedures in future practise.

*‘Some of the hazards highlighted in the resource show how dangerous it (cannulation) is… haematomas and problems that can be caused by that. So that was one aspect that I will build on in the future.’*

The interviews showed that the process helped students to feel more prepared for F1 posts by highlighting basic human factors principles and safety behaviours that they can integrate in their practise.

*‘I think it has just reinforced some of the basic practises that we have got lazy in and forget… Making sure that we keep on going in August (as F1 doctors), makes us more safe practitioners going on.’*

*‘I definitely feel it has helped prepare me more for the job of cannulation when I’m an F1.’*

## Discussion

We found improvements in safety behaviours as a result of the e-learning training, which were related to various components of a healthcare socio-technical system, as highlighted by the SEIPS model [24]. The major systems impacted by the e-learning were found to relate to ‘environment’, ‘person’, ‘organisation’ and ‘tasks’, while there appeared to be less impact on ‘tool’ factors. We anticipated 90–120 min to complete all four modules, so were surprised to find higher average engagement with the e-learning. This finding, when triangulated with the results from VRE and interviews, suggests that the e-learning was a major source of behavioural change. This study was an exploratory study into the use hybrid simulation and VRE to assess behaviour change following e-learning training in human factors. As such, there were no control groups to compare human factors training by other methods.

Demonstrative videos with interactive elements were a key pedagogical influence for these changes. This aligns with evidence that passive guidance through a resource, followed by interactive, retrospective activities, maximises the learning potential from demonstrative videos [29–31]. The impact of demonstrative videos has only been evidenced previously for communication and teamworking skills [20, 32]. We propose that demonstrative videos with interactive exercises can have impacts on broader human factor-related behaviours. E-learning has limitations with regards to improving psychomotor skills when used in isolation [33], thus the blending of e-learning with real practise or simulation is more likely to influence safety behaviours [33, 34]. Using e-learning as an adjunct to simulation training improves the cost-efficiency of training than using more than simulation alone [7, 35]. We used hybrid simulation as a method to assess change in behaviour following e-learning on intravenous cannulation skills and human factors. Combining hybrid simulation with VRE, to encourage learner reflection on their performance, allowed the students to self-debrief after analysing their own videos. There is equivocal evidence in the literature around the benefit of video-assisted debriefing for team-based simulation [36]. However, we found self-debriefing with a VRE template to provide a valuable method for self-assessment on behaviour change related to the SEIPS model, when performing intravenous cannulation in a challenging simulation scenario.

The VRE responses and interviews demonstrated impacts of the e-learning across four different themes. Control of environmental human factors is a simple but important concept that our participants had not previously considered and would change in their practise going forward. The training delivers basic physical ergonomic awareness, which encourages manipulation of the working environment to increase the chance of success, and decrease scope for human error [37].

From a cognitive ergonomics perspective, empowerment and education of the patient were a crucial practise that students began to apply following training. A systematic review and meta-analysis both concluded that such behaviour leads to improved patient satisfaction, treatment adherence and outcomes [38, 39]. Improved documentation was found to be another major impact of the training. The interviews demonstrated improvements in awareness and operation of documentation—in completion and quality. This was evidenced on the ward with positive nurse feedback, suggestive of level three Kirkpatrick evaluation [40]—behaviour change in the workplace—which would indicate high impact from the e-learning resource.

Participants reported feeling more prepared for practise, with a greater awareness of safety issues. This may have positive effects on overall safety culture relating to staff attitudes, perceptions and patterns of behaviour [41], which are known to impact upon patient and staff outcomes [42].

There have been repeated calls in the literature to accelerate the integration of human factors and ergonomics principles in healthcare [37, 43]. A core building block to achieving this is awareness and understanding of human factors principles. We have shown added value in preparing medical students for practise by incorporating human factors training into the training of a key clinical skill for foundation doctors [2, 23]. Guidance from the General Medical Council (GMC) [2] and the World Health Organization (WHO) [4] suggests that human factors should be taught at undergraduate level. Human factors and ergonomics training could be given higher status in undergraduate studies by embedding such training in curricula. We have found e-learning to be an effective tool to do this alongside more established methods such as team-based simulation as as part of a *Simulation Augmented Medical Education* pedagogy advocated by Norman [7].

## Strengths and limitations

Using the SEIPS model [24] to guide and frame systems interactions at an individual level focused the participants and results on human factors behaviours. The combined VRE alongside semi-structured interviews with all ten participants led to a rich data-set. To formulate interview questions, analyse data and develop themes, the research team worked together, reducing the risk of bias and enhancing credibility. The study used a convenience sample from a single year group in one medical school, which may have limited the overall trustworthiness of results. In addition, the sample may not truly represent final-year medical students and may have been biased by those interested in the study or from unknown demographics. Improvements in performance could also be attributed to repetition of a similar scenario.

Long-term follow-up or clinical observation could have strengthened the findings. Indications towards Kirkpatrick level three [40] evaluation were unexpected and suggest high impact of the e-learning intervention. This work was carried out in one cohort of final-year medical students concentrating on a single task (peripheral intravenous cannulation). However, incorporating human factors training into e-learning for specific procedural skills could be beneficial for undergraduate, postgraduate and higher-level speciality training.

### Future work

We found hybrid simulation combined with VRE to be informative in assessing behaviour change following e-learning on human factors but longitudinal studies are required to assess long-term impacts of the training in the working environment. VRE has been shown to be a suitable method of enquiry to develop deeper understandings of the complex nature of clinical work and make tacit knowledge explicit. VRE techniques could be used for future training resources or systems design [44]. Further, studies including controlled trials comparing different methods of training in human factors would be useful.

## Conclusion

The study evidences the value of e-learning—particularly demonstrative videos with interactive activities for human factors training in undergraduate medical students. E-learning is a valuable method by which the human factors learning outcomes for medical graduates, recommended by the GMC [2] and WHO [4], can be achieved. The use of e-learning for training in intravenous cannulation skills and human factors has demonstrated impact on students’ preparedness for practise and ongoing clinical performance. Used in undergraduate training, it can support students’ transition to becoming a doctor.

## Supplementary information

**Additional file 1: Appendix 1.** Reflective template

## Data Availability

The datasets used and/or analysed during the current study are available from the corresponding author on reasonable request.

## Abbreviations

BD: Becton Dickinson
CRM: Crisis Resource Management
F1: Foundation year one doctor
GMC: General Medical Council
PTT: Part-Task Trainer
SEIPS: The Systems Engineering Initiative for Patient Safety model
SP: Standardised patient
VRE: Video-reflexive ethnography
WHO: World Health Organization
