# Supplementary material for: A qualitative study using hybrid simulation to explore the impacts of human factors e-learning on behaviour change
Source: Adv Simul (Lond). 2020 Aug 12;5:20. doi: 10.1186/s41077-020-00136-y (PMC7425130; doi:10.1186/s41077-020-00136-y)
Supplement: Supplementary file 1 — Additional file 1: Appendix 1. Reflective template [file 41077_2020_136_MOESM1_ESM.docx]

#### **Appendix 1**

#### Reflective Template

Now that you have completed both simulated scenarios and have access to your filmed performances, we ask you to type some reflective comments based on your performances.

Once you have finished writing the reflection, please send a copy to ***** and review your response before the interview ready for discussion.

Please answer the question below using the framework that we have provided (no more than 100 words per heading).

**Question:** When comparing your footage of simulation 1 and 2, what things did you do differently in relation to:

1. The workspace? (E.g. Lighting, organising your workspace, gathering equipment, patient/inserter position)
2. The tools you used? (E.g. The cannula, tourniquet, cleaning wipes, sharps bin, dressings and familiarity with the equipment)
3. The tasks you performed? (E.g. Cannulation, informed consent, documentation, monitoring)
4. The people you interacted with? (E.g. Patient support, interaction with the team and patient, shared decision making, giving information)
5. The organisation? (E.g. Following policy, dealing with team culture and poor organisational design)
6. Other:

Thank you for your cooperation.
